# Aligned nanofibers in biomimetic periosteal extracellular matrix/poly(ε-caprolactone) membranes enhance bone regeneration via the ITGB1/PI3K/AKT pathway

**DOI:** 10.1093/rb/rbaf099

**Published:** 2025-09-29

**Authors:** Zhuohao Wen, Shuyi Li, Huiguo Qiu, Xueyan Liu, Zhiying You, Yuhan Yan, Yuejuan Che, Miao Zhou

**Affiliations:** Department of Stomatology, Guangdong Provincial People’s Hospital (Guangdong Academy of Medical Sciences), Southern Medical University, Guangzhou 510080, China; Department of Stomatology, Guangdong Provincial People’s Hospital (Guangdong Academy of Medical Sciences), Southern Medical University, Guangzhou 510080, China; Zhuhai Stomatological Hospital, Zhuhai 519000, China; Affiliated Stomatology Hospital of Guangzhou Medical University, Guangdong Engineering Research Center of Oral Restoration and Reconstruction, Guangzhou Key Laboratory of Basic and Applied Research of Oral Regenerative Medicine, Guangzhou 510182, China; Affiliated Stomatology Hospital of Guangzhou Medical University, Guangdong Engineering Research Center of Oral Restoration and Reconstruction, Guangzhou Key Laboratory of Basic and Applied Research of Oral Regenerative Medicine, Guangzhou 510182, China; Department of Stomatology, Huadu District People’s Hospital of Guangzhou, Southern Medical University, Guangzhou 510800, China; Department of Stomatology, Guangdong Provincial People’s Hospital (Guangdong Academy of Medical Sciences), Southern Medical University, Guangzhou 510080, China; Department of Stomatology, Guangdong Provincial People’s Hospital (Guangdong Academy of Medical Sciences), Southern Medical University, Guangzhou 510080, China; Department of Biomedical Engineering, School of Materials Science and Engineering, South China University of Technology, Guangzhou 510641, China; Department of Anesthesiology, Sun Yat-sen Memorial Hospital, Sun Yat-sen University, Guangzhou 510120, China; Department of Stomatology, Guangdong Provincial People’s Hospital (Guangdong Academy of Medical Sciences), Southern Medical University, Guangzhou 510080, China

**Keywords:** biomimetic periosteum, topological morphology, periosteal extracellular matrix, bone regeneration, integrin β1

## Abstract

Periosteum plays an indispensable role in bone regeneration by providing osteogenic and angiogenic cues essential for tissue repair. In cases of severe bone defects or nonunion, autologous vascularized periosteum transplantation remains a highly effective clinical solution. However, its application is restricted by donor site morbidity and limited tissue availability, thereby underscoring the urgent need for artificial periosteum that mimics both the composition and structure of the native counterpart. Among these properties, the topological morphology of the periosteum is believed to be critical, yet its influence on bone regeneration remains insufficiently understood. In this study, biomimetic periosteum membranes composed of coaxially electrospun poly(ε-caprolactone) (PCL) and periosteal extracellular matrix (pECM) were fabricated with either random or aligned nanofiber architectures. Their osteogenic potential was systematically evaluated *in vitro* and *in vivo*. Compared to the randomly arranged structure, aligned pECM (aPEC) significantly enhanced the adhesion, alignment, and osteogenic differentiation of bone marrow mesenchymal stem cells (BMSCs) by activating the ITGB1/PI3K/AKT signaling pathway, whereas these effects were not observed in pure PCL membranes. These findings demonstrate that aligned topological morphology in biomimetic periosteum plays a pivotal role in directing stem cell behavior and promoting bone regeneration. This work provides mechanistic insight and technical guidance for the future design of functionally enhanced artificial periosteum for bone tissue engineering applications.

## Graphical abstract

**Figure:**
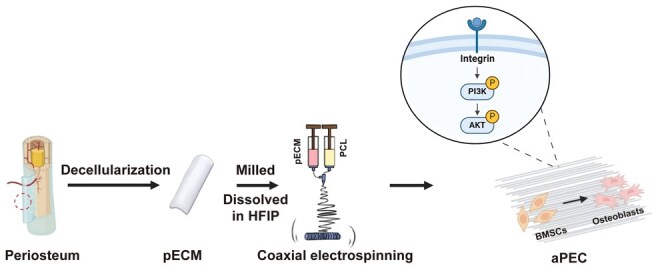

## Introduction

The periosteum is a fibrous tissue that covers the surface of cortical bone. It serves as a reservoir of cells for bone regeneration and promotes both osteogenesis and vascularization [1]. Additionally, the periosteum plays a crucial role in bone regeneration and remodeling. The loss of the periosteum can lead to a significant decrease in new bone formation and a 10-fold reduction in vascularization, thus impairing bone regeneration [2].

Several commercial periosteum substitutes and guided bone regeneration membranes, such as bioresorbable collagen membranes (e.g. Bio-Gide^®^) and synthetic polymer-based membranes (e.g. PLA and PLGA), have been developed to support bone healing in clinical applications [3–5]. Although these products are widely used in periodontal and orthopedic surgeries, their limited bioactivity, suboptimal mechanical properties, and inability to replicate the native periosteum’s anisotropic architecture significantly constrain their efficacy in promoting vascularized bone regeneration [6, 7].

Recent studies have demonstrated that periosteum exhibits an aligned topological structure, with cells adopting an elongated spindle-shaped morphology within neatly arranged collagen fibers, consistent with osteoblast differentiation on the cortical bone surface [8]. This suggests that the periosteum’s topological structure facilitates osteogenesis by modulating cell behavior. However, the role of periosteal topological morphology in regulating osteoblast behavior remains controversial. Previous studies have examined artificial periosteum fabricated from synthetic polymers, including poly(ε-caprolactone) (PCL), polylactic acid (PLA), and poly(lactic-co-glycolic acid) (PLGA), as well as natural materials such as type I collagen. However, findings remain inconsistent. Some studies report that topological features, such as aligned nanofibers, can guide cell orientation and promote osteogenic differentiation [9–11], whereas others show no significant differences compared to random fiber scaffolds [12–15]. Moreover, most scaffolds lack periosteum-derived extracellular matrix components or fail to replicate the anisotropic microarchitecture of natural periosteum, limiting insights into how periosteal topological cues regulate bone regeneration.

To date, biomimetic periosteum derived from natural components to replicate its oriented topological structure has not yet been developed. Furthermore, most studies emphasize the influence of material microstructure on cell behavior, with limited *in vivo* analyses and mechanistic insights. In our previous work, a biomimetic periosteum derived from natural porcine periosteum was developed using PCL/periosteal extracellular matrix (pECM) coaxial electrospun membranes (PEC), with the pECM on the outer surface preserving biological activity [16]. However, the effect of fiber alignment on topological morphology and its regulation of osteogenic processes has not been systematically examined.

**Figure 1.**
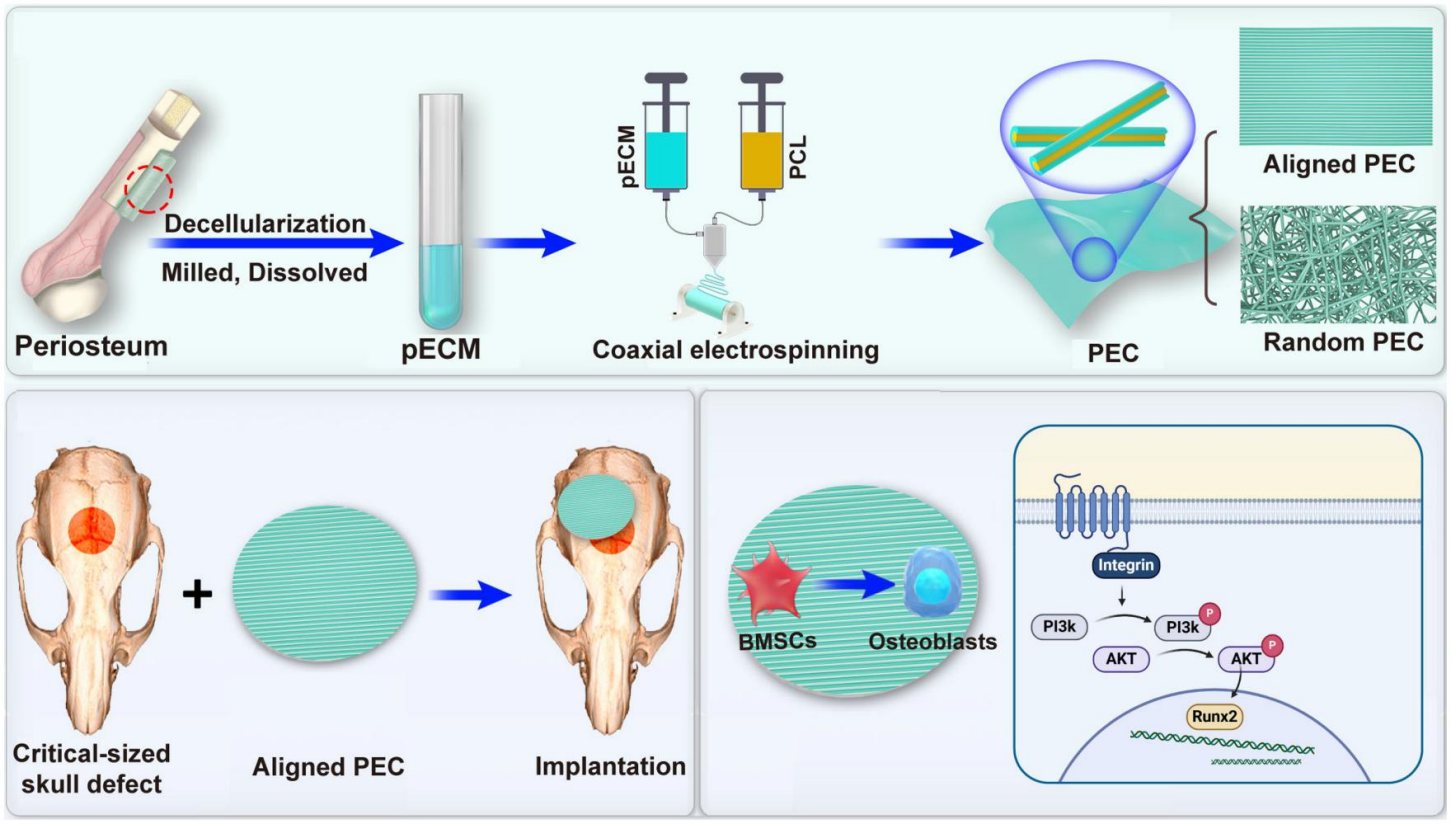
Electrospun membranes with random and aligned pECM structures were fabricated via coaxial electrospinning. The aligned pECM topology enhanced BMSC adhesion and osteogenic differentiation via the ITGB1/PI3K/AKT signaling pathway, ultimately facilitating bone regeneration.

To further investigate this topic, aligned pECM were fabricated using established coaxial electrospinning techniques to replicate the composition and topology of natural periosteum, and their effects on the cytological behavior of bone marrow mesenchymal stem cells (BMSCs) were evaluated. The oriented topological structure of the periosteum was shown to enhance the adhesion and osteogenesis of BMSCs *in vitro* and *in vivo*. Furthermore, this regulatory effect was mediated via the ITGB1/PI3K/AKT signaling pathway (Figure 1). This study provides a novel model and valuable insights into periosteum topological morphology, establishing a theoretical foundation for the development of biomimetic, tissue-engineered periosteum.

## Materials and methods

### Obtaining and decellularizing porcine periosteum

Fresh femurs from 6-month-old Yorkshire pigs were obtained from a local slaughterhouse, and the overlying muscle tissue was carefully removed to expose the periosteum. The periosteum was stripped from the femur using a periosteal stripper, washed with ultrapure water, frozen at −80°C for 6 h, thawed in a 37°C water bath for 30 min, and subjected to three freeze-thaw cycles before being decellularized under sterile conditions. The tissues were treated with 1% Triton X-100 for 10 h, followed by 1% sodium dodecyl sulfate (SDS) for 2 h, and 100 U/mL DNase in a 37°C water bath overnight. Finally, the tissues were rinsed 3× with ultrapure water.

### Characterization of pECM and detection of DNA content

To evaluate the decellularization efficiency, pECM was fixed in 10% neutral formalin solution for 24 h, followed by programmed dehydration and paraffin embedding. The samples were sectioned into 5 μm thick slices using a microtome, followed by hematoxylin and eosin (HE), Masson, and DAPI staining. HE and Masson's trichrome staining were examined under optical microscopy, while DAPI staining was observed using confocal microscopy. To quantify residual DNA content, 35 mg of pECM was freeze-dried, fragmented, and subjected to genomic DNA extraction using a DNA extraction kit. The extracted DNA was then quantified using the Quant-iT PicoGreen dsDNA kit (Invitrogen, USA) (*n* = 3).

### Preparation of PEC

The pECM was freeze-dried, crushed, and sieved using a 40-mesh screen. The resulting material was further ground in an automatic freeze grinder (parameters: −10°C, 60 Hz, 300 s) to produce pECM powder with a particle size smaller than 425 μm. A total of 500 mg of the pECM powder was dissolved in 10 mL of hexafluoroisopropanol (HFIP, Aladdin, China) and magnetically stirred at 4°C for 6 days to produce a 5% (w/v) shell solution. Similarly, 1.2 g of PCL particles (molecular weight: 80 000) were dissolved in 10 mL of HFIP to prepare a 12% (w/v) PCL core solution. For PEC membrane fabrication, the prepared solutions were loaded into two 10 mL syringes attached to syringe pumps. Coaxial electrospinning was carried out using a coaxial nozzle (outer diameter: 16 G, inner diameter: 22 G) with a constant extrusion rate of 1 mL/h for the PCL solution, a gradient voltage of 8–12 kV, and a gradient extrusion rate of 1.2–2.0 mL/h for the pECM solution. The electrospinning process was conducted at 25°C and 40–50% relative humidity, with fibers collected 10 cm from the spinneret on a metal roller collector covered with aluminum foil. The collector rotated at 100 rpm to produce random morphology PEC (rPEC) and at 3200 rpm for aPEC Additionally, random morphology PCL (rPCL) and aligned PCL (aPCL) were fabricated as controls. The prepared membrane was soaked in 75% ethanol for 3 h and subsequently sterilized using UV irradiation for 4 h.

### Characterization of PEC

The dried electrospun membrane was trimmed to appropriate sizes and mounted onto a gold-sputtering stage. Following gold sputtering, the fiber morphology was examined using a scanning electron microscope (SEM, Hitachi, Japan). Orientation analysis of the SEM images was performed with ImageJ software. To observe the core-shell structure, PEC fibers were collected on a 230-mesh carbon support membrane and imaged using a transmission electron microscope (TEM, JEOL, Japan). The water contact angle of the electrospun membrane was measured using an OCA20 contact angle meter (*n* = 3).

### Characterization of chemical composition

X-ray photoelectron spectroscopy (XPS) and Fourier-transform infrared (FTIR) spectroscopy were performed to analyze the surface chemical composition of the membranes.

FTIR spectroscopy was performed using a Thermo Scientific Nicolet iS50 FTIR Micro Spectrometer (Thermo Fisher Scientific, USA) in attenuated total reflectance (ATR) mode. Spectra were recorded over the range of 4000–400 cm^−1^ with a resolution of 4 cm^−1^ and averaged over 32 scans.

XPS analysis was performed using a Thermo Scientific Nexsa G2 Micro-focus XPS system (Thermo Fisher Scientific, USA). Spectra were acquired with a monochromatic Al Kα X-ray source (hν = 1486.6 eV) operated at 72 W (12 kV × 6 mA) under a base pressure of <10^−7  ^Pa. Survey scans were collected over the range of 0–1350 eV with a pass energy of 200 eV, while high-resolution spectra of N 1 s were obtained at a pass energy of 50 eV for detailed chemical state analysis. Charge neutralization was applied during measurement, and binding energies were calibrated using the C 1 s peak at 284.8 eV as reference.

### Mechanical properties evaluation

Tensile mechanical testing was conducted using a universal testing machine (Instron 68TM-10, Instron, USA) to evaluate the stress–strain behavior of the membranes. Owing to the anisotropic structure of aPEC, specimens were prepared in two orientations: parallel to the fiber alignment (y-direction) and perpendicular to the fiber alignment (x-direction). Dog-bone-shaped samples (dimensions: 6 ×35 mm, gauge length: 10 mm) were stretched at a crosshead speed of 10 mm/min until failure. The ultimate tensile strength and elongation at break were calculated from the stress–strain curves. All tests were conducted at room temperature in triplicate for each orientation.

### Physicochemical stability evaluation

Swelling behavior of the membranes was evaluated by immersing preweighed dry samples in PBS at 37°C for predetermined time points (5 min, 30 min, 1 h, 6 h, 12 h, and 24 h). At each time point, the samples were removed (*n* = 3), gently blotted to remove surface moisture, and weighed. The swelling ratio (%) was calculated as: (*W_s_* − *W_d_*)/*W_d_ *× 100%, where *W_s_* and *W_d_* are the weights of the swollen and dry samples, respectively.

*In vitro* degradation was assessed by incubating the membrane samples in PBS at 37°C for 30 days. At 5-day intervals (5, 10, 15, 20, 25, and 30 days), the samples were retrieved, rinsed with deionized water, lyophilized, and weighed. The remaining mass (%) was calculated as: (*W_t_*/*W*_0_) × 100%, where *W_t_* is the dry weight at time *t*, and *W*_0_ is the initial dry weight.

### CCK-8 assay

Mouse BMSCs were purchased from Wuhan Procell Company and cultured in Dulbecco’s Modified Eagle Medium (DMEM; Gibco, USA) supplemented with 10% fetal bovine serum (FBS; ExCell Bio, China) and 1% penicillin-streptomycin (PS; Gibco, USA) at 37°C in a humidified atmosphere with 5% CO_2_. The cell counting kit-8 (CCK-8) assay was performed to evaluate the effect of electrospun membranes on BMSC proliferation. The study included four groups: (i) rPCL, (ii) aPCL, (iii) rPEC, and (iv) aPEC. Electrospun membranes from each group were fixed at the bottom of 24-well plates (*n* = 5), and BMSCs were seeded onto the membrane surfaces. After cell adhesion, 500 μL of serum-free medium was added to each well, followed by 50 μL of CCK-8 solution (Beyotime, China) at 24, 48, and 72 h. After 2 h of incubation, the culture medium was transferred to a 96-well plate, and the optical density (OD) at 450 nm was measured using a microplate reader (Thermo, USA).

### Live/dead staining

Live/dead staining was conducted to evaluate the cytocompatibility of the electrospun membranes. Electrospun membranes were fixed to the bottom of 24-well plates as per the described groupings (*n* = 3), followed by seeding and culturing of BMSCs for 72 h. After removing the medium, cells were washed with phosphate-buffered saline (PBS; Gibco, USA). Subsequently, 300 μL of Calcein AM/PI staining solution (Beyotime, China) was added to each well, and the plates were incubated at 37°C in the dark for 30 min. The stained cells were then observed under a fluorescence microscope.

### ALP and ARS staining

BMSCs were seeded on the electrospun membranes fixed at the bottom of 24-well plates, grouped as described (*n* = 3). The cells were cultured in an osteogenic induction medium, with the medium replaced every 2 days. Alkaline phosphatase (ALP) staining was performed on day 7. Briefly, cells were fixed with 4% paraformaldehyde for 20 min, washed 3× with PBS, and incubated with BCIP/NBT solution (Beyotime, China) at room temperature in the dark for 30 min. After incubation, the cells were washed 3× with PBS and observed under a stereo microscope.

Alizarin red S (ARS) staining was conducted on day 14 (*n* = 3). Cells were fixed with 4% paraformaldehyde for 20 min, washed 3× with PBS, and stained with ARS solution (Beyotime, China) at room temperature for 30 min. The staining results were observed and imaged under a stereo microscope. To quantify calcium nodule deposition, 10% cetylpyridinium chloride (CPC) solution was added to dissolve the calcium nodules, and the OD value at 562 nm was measured using a microplate reader (Thermo, USA).

### ALP activity detection

BMSCs were cultured on electrospun membranes fixed at the bottom of six-well plates, grouped as described (*n* = 3). After 7 days of culture, 150 µL of RIPA lysis buffer (Beyotime, China) was added to each well, and the cells were lysed on ice for 30 min. The lysate was collected and centrifuged at 12 000 rpm for 20 min at 4°C, and the supernatant was retained while discarding the precipitate. The total protein concentration in the samples was quantified using a BCA protein assay kit (Beyotime, China). Subsequently, ALP activity was determined and calculated using an alkaline phosphatase detection kit (Beyotime, China) according to the manufacturer’s instructions.

### Western blot

BMSCs were seeded onto electrospun membranes fixed at the bottom of six-well plates, grouped as described (*n* = 3). After 7 days of culture, cells were lysed with RIPA lysis buffer, and SDS-PAGE loading buffer was added to the lysate. The 7-day time point was selected because it represents a critical stage in osteogenic differentiation, during which early (e.g., BMP-2) and late (e.g. OPN) markers begin to exhibit detectable expression levels [17–20].

The mixture was then boiled in a 95°C metal bath for 10 min. Proteins were separated by 10% SDS-PAGE and transferred onto a polyvinylidene fluoride (PVDF) membrane. The membrane was blocked using a blocking buffer (Beyotime, China) for 15 min and subsequently incubated with anti-Runx2, anti-BMP-2, anti-OPN, and anti-GAPDH primary antibodies (Proteintech, China) at 4°C overnight. After washing, the membrane was incubated with a secondary antibody at room temperature for 1 h on a shaker. Protein expression levels were visualized using the Odyssey CLx imaging system (Biosciences, USA), and the relative band densities were semiquantified using ImageJ software.

### Animal experiment

Healthy 8-week-old male SPF Sprague-Dawley rats were obtained from the Guangdong Medical Experimental Center. All experimental procedures were approved by the Experimental Animal Ethics Committee of Guangdong Huawei Testing Co., Ltd. (No. HWT-BG-117b). The study included five groups: (i) blank group; (ii) rPCL group; (iii) aPCL group; (iv) rPEC group; and (v) aPEC group (*n* = 6). The bone repair effect was evaluated using a bilateral critical-size skull defect model.

Before surgery, the skull hair was removed, and the skin was disinfected. General anesthesia was administered via intraperitoneal injection of sodium pentobarbital. A midline incision was made, and the skin, muscle, and periosteum were bluntly separated to expose the skull. Using a 5 mm diameter trephine, full-thickness bone defects were created on both sides of the skull, ensuring the integrity of the dura mater. The membranes inoculated with BMSCs were placed over the defect sites according to the grouping, while no material was implanted in the blank group. The wounds were closed in layers using sutures.

Four weeks post-implantation, all animals were euthanized with isoflurane. Skull specimens were collected and fixed in 4% paraformaldehyde for 24 h. Microcomputed tomography (Micro-CT; SkyScan, Belgium) was performed to image the specimens. Scanning parameters included: a 1 mm thick aluminum filter, spatial resolution of 2000 × 1332, slice thickness of 18 μm, current of 200 μA, and voltage of 85 kV. The acquired data were processed using NRecon 1.0 software to define the volume of interest (VOI) within the bone defect area. The threshold for new bone formation was set to 70–255 using CTAn 1.13 software, and the bone volume-to-total volume ratio (BV/TV) was calculated.

Finally, the specimens were subjected to HE and Masson staining, following the same protocols as described earlier.

### Immunofluorescence staining

To evaluate cell adhesion, BMSCs were seeded on rPEC or aPEC membranes fixed at the bottom of a 24-well plate (*n* = 3). After 12 h of culture, the cells were fixed with 4% paraformaldehyde for 15 min and washed 3× with PBS. Permeabilization was performed using a PBS solution containing 0.1% Triton X-100. Actin-Tracker (Beyotime, China) was subsequently added, and the samples were incubated at room temperature in the dark for 50 min. Following incubation, the cells were washed 3× with PBS containing 0.1% Triton X-100. The cytoskeleton was visualized using confocal microscopy.

### Transcriptome sequencing and bioinformatics analysis

To investigate the differential RNA transcript expression in BMSCs between the rPEC and aPEC groups, whole-genome transcriptome sequencing was conducted by Guangdong Magigene Biotechnology Co., Ltd. Differential expression analysis was performed using the DESeq2 R package (*n* = 4). Genes with a *P* value <0.05, as determined by DESeq2, were considered statistically significant. Additionally, Kyoto Encyclopedia of Genes and Genomes (KEGG) pathway enrichment analysis and Gene Ontology (GO) enrichment analysis were conducted to explore the biological functions and pathways involved.

To validate the activation of signaling pathways, the expression levels of integrin β1 (ITGB1), p-PI3K, PI3K, p-AKT, AKT, and GAPDH in the rPEC and aPEC groups were measured through WB analysis, following the previously described protocol.

### ITGB1 inhibition assay

To investigate whether the osteogenic effects of aPEC are mediated through ITGB1 signaling, BMSCs were seeded onto rPEC and aPEC membranes fixed at the bottom of six-well plates and cultured under osteogenic induction conditions. After cell adhesion, the medium was supplemented with the ITGB1 inhibitor ATN‑161 (20 μM; Selleck, TX, USA) in the experimental groups, while the corresponding control groups were cultured in osteogenic induction medium without ATN‑161. The cells were maintained under these conditions for 7 days, with medium refreshed every 2 days. At the end of the treatment, cells were lysed for WB analysis to evaluate the expression of ITGB1, p‑PI3K, PI3K, p‑AKT, AKT, RUNX2, and BMP‑2. Other procedures were performed as described above.

### Statistical analysis

All experimental data were presented as mean ± standard deviation (SD). Statistical analyses were conducted using GraphPad Prism software. Comparisons between two groups were evaluated using Student’s *t*-test, while differences among three or more groups were assessed by one-way analysis of variance (ANOVA) followed by Tukey’s *post hoc* test. Statistical significance levels were defined as ***P < 0.001, **P < 0.01, and *P < 0.05.

## Results

### Construction of aligned pECM

**Figure 2.**
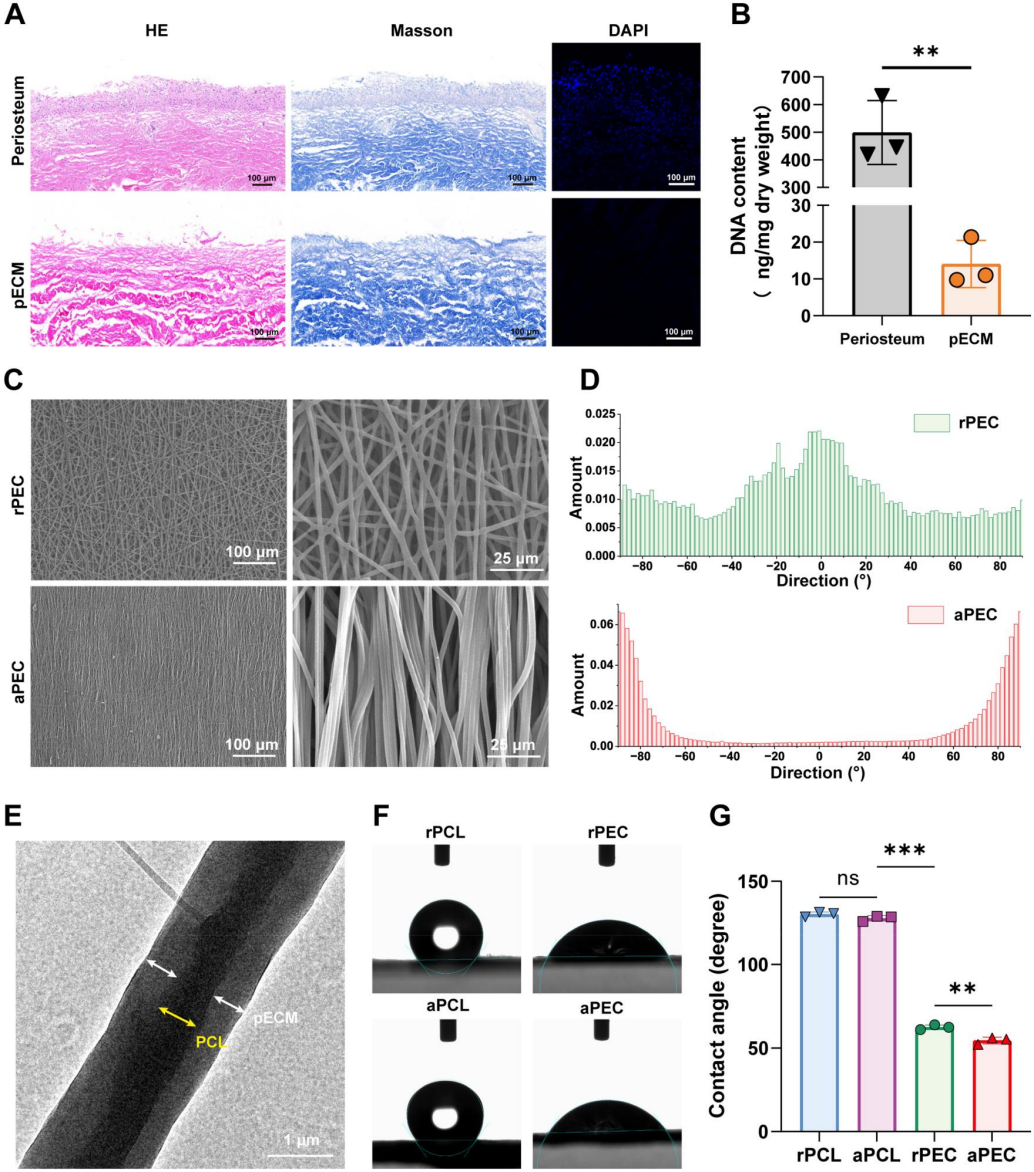
Construction of aligned pECM. (**A**) HE, masson, and DAPI staining of the periosteum and pECM samples. (**B**) DNA content analysis of the periosteum and pECM. (**C**) SEM images of random and aligned PEC fibers, and (**D**) their corresponding fiber angle distribution with respect to the horizontal. (**E**) Transmission electron microscopy highlighting the ‘core-shell structure’ with yellow arrows pointing to PCL cores and white arrows to pECM shells. (**F**) Water contact angle measurements for PCL and PEC fibers, with (**G**) presenting their quantitative analysis. Data are presented as mean ± SD. ns, no significance, ***P < 0.001, **P < 0.01, *P < 0.05.

HE and DAPI staining confirmed the lack of visible nuclear material in pECM. Masson staining revealed that pECM retained the collagen component of the periosteum and exhibited a loose, multilayered structure (Figure 2A). The DNA content of native periosteum was 498.90 ± 115.60 ng/mg, which decreased to 14.04 ± 6.39 ng/mg after decellularization (Figure 2B), meeting the decellularized tissue standard (DNA content < 50 ng/mg).

The surface microstructures of random and aligned fiber membranes are shown in Figure 2C. Electrospun fibers in the rPEC group were randomly arranged, forming a multiangle network structure. In contrast, fibers in the aPEC group exhibited an aligned arrangement with horizontal distribution angles concentrated between 80° and 90° (Figure 2D). TEM revealed that the pECM shell encapsulated a high-density PCL core, forming a distinct coaxial structure (Figure 2E).

Water contact angle measurements indicated that the hydrophilicity of PEC was significantly enhanced compared to PCL due to the pECM coating (*P* < 0.001). The water contact angle of the aPEC group (54.53 ± 2.06°) was lower than that of the rPEC group (62.47 ± 1.40°). However, no significant differences were observed between the aPCL group (127.70 ± 1.69°) and the rPCL group (130.10 ± 1.39°) (Figure 2F–G).

### Characterization of aPEC

**Figure 3.**
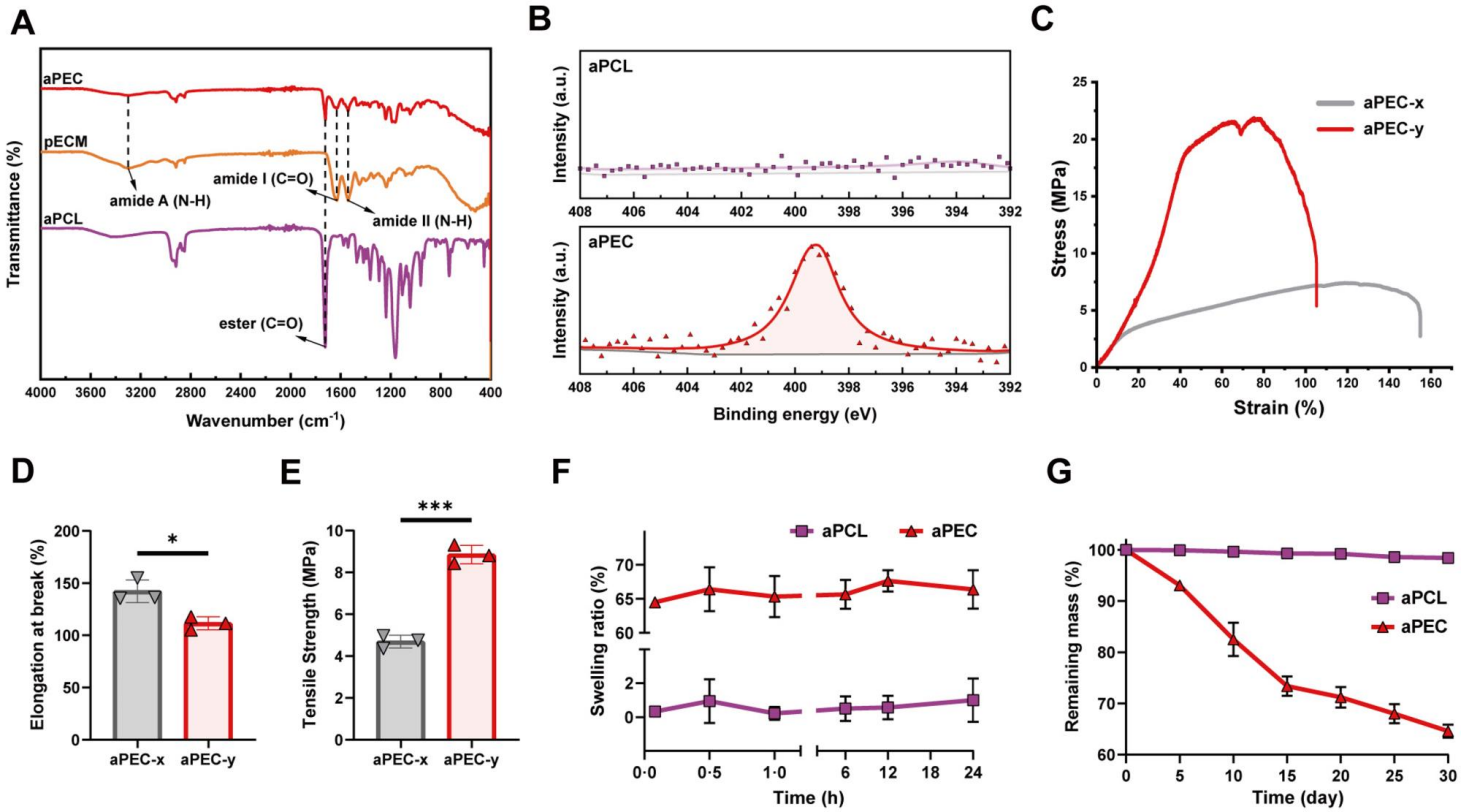
Characterization of aPEC. (**A**) FTIR spectra of aPCL, pECM, and aPEC. (**B**) High-resolution XPS N 1s core-level spectra of aPCL and aPEC. (**C**) Stress–strain curves of aPEC membranes measured parallel (aPEC‑y) and perpendicular (aPEC‑x) to the fiber alignment. (**D**) Elongation at break of aPEC in the x- and y-directions. (**E**) Tensile strength of aPEC in the x- and y-directions. (**F**) Swelling ratios of aPCL and aPEC membranes over 24 h. (**G**) Degradation profiles showing the remaining mass of aPCL and aPEC after 30 days. Data are presented as mean ± SD. ns, no significance, ***P < 0.001, **P < 0.01, *P < 0.05.

The FTIR spectra of aPCL, pECM, and aPEC are presented in Figure 3A. The PCL spectrum exhibits a characteristic absorption peak at 1725 cm^−1^, corresponding to the stretching vibration of the ester carbonyl group (C = O) in polycaprolactone. Distinct protein-related peaks are observed in the pECM spectrum, including amide A (3300 cm^−1^, N–H stretching vibration), amide I (1640 cm^−1^, C = O stretching vibration), and amide II (1540 cm^−1^, N–H bending and C–N stretching vibration). In the PEC spectrum, the characteristic peaks of both PCL and ECM are simultaneously detected, particularly the amide A, I, and II peaks, further confirming that ECM was successfully coated on the surface of PCL fibers. These findings indicate that ECM was immobilized on the PCL surface, with the binding likely mediated by hydrogen bonding and physical adsorption between ECM components and the PCL matrix.

High-resolution N 1 s XPS analysis (Figure 3B) revealed no nitrogen signal in the aPCL fibers, consistent with the chemical composition of PCL. In contrast, a prominent N 1 s peak at 399.5 eV was detected in aPEC fibers, attributed to the nitrogen (N–C = O) of the amide groups present in ECM proteins. These results provide direct evidence of the successful coating of PCL nanofibers with pECM.

The tensile stress–strain curve of aPEC is shown in Figure 3C. The direction parallel to the oriented fiber alignment was defined as the Y-axis, whereas the direction perpendicular to it was defined as the X-axis. When stretched along the Y-axis, the oriented membrane exhibited a higher maximum tensile strength (8.86 ± 0.44 MPa) and a lower elongation at break (111.50 ± 6.26%). In contrast, stretching along the X-axis resulted in superior ductility, with a maximum tensile strength of 4.69 ± 0.31 MPa and an elongation at break of 142.20 ± 10.96% (Figure 3D and E**)**. Notably, the tensile strength of aPEC in both directions exceeded that of native periosteum (approximately 3–4 MPa), suggesting that aPEC exhibits favorable mechanical properties for use as an artificial periosteum [21, 22].

As shown in Figure 3F, aPCL displayed a minimal swelling ratio (1.00 ± 1.28%), indicative of its hydrophobic nature and poor water absorption capacity. By contrast, aPEC showed a substantially higher swelling ratio (66.37 ± 2.82%), attributed to the pECM components imparting superior hydrophilicity. Notably, aPEC rapidly absorbed water, achieving 64.51 ± 0.17% swelling within 5 min, and maintained stable swelling behavior over the following 24 h.

The degradation behavior of the materials is presented in Figure 3G. aPCL exhibited negligible degradation over the 30-day observation period, with the remaining mass consistently above 98%. In contrast, aPEC displayed a gradual mass loss, retaining 64.6 ± 1.28% of its original mass after 30 days. The controlled degradability of aPEC is attributed to the presence of pECM components, which enhance hydrophilicity and promote hydrolytic degradation.

### pECM promote BMSC proliferation

**Figure 4.**
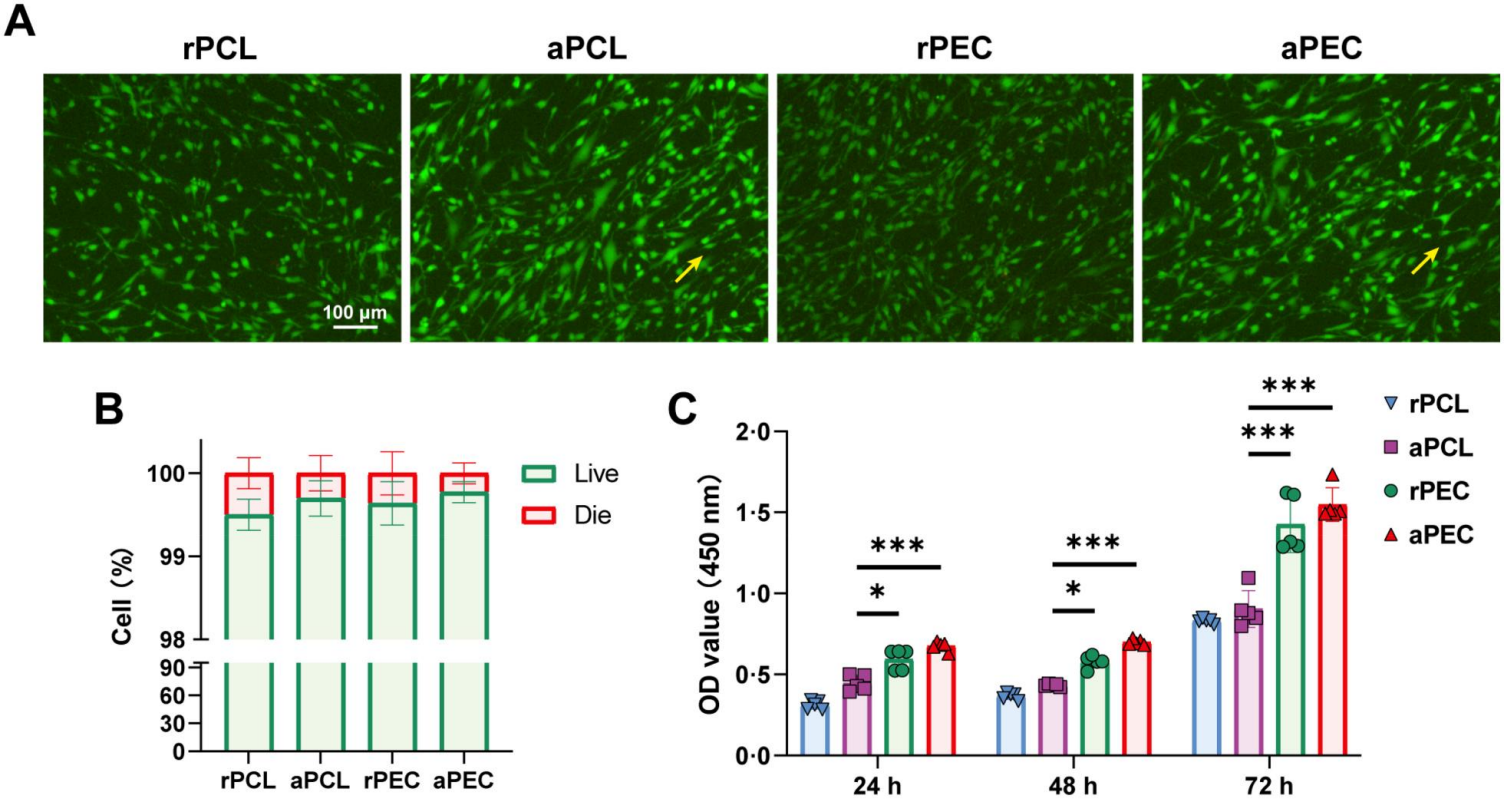
pECM promote BMSC proliferation. (**A**) Live/dead staining to evaluate membrane cytocompatibility, with green (calcein-AM) marking living cells and red (PI) marking dead cells; yellow arrows show the alignment direction of the electrospun fibers. (**B**) Quantitative analysis of live/dead staining. (**C**) CCK-8 assay showing the proliferation of BMSCs on random and aligned electrospun membranes. Data are presented as mean ± SD. ns, no significance, ***P < 0.001, **P < 0.01, *P < 0.05.

To confirm the absence of harmful residues in the electrospun membranes, live/dead staining was conducted. Fluorescence microscopy images revealed predominantly green-stained live cells (>99%), with no red-stained dead cells across all groups, confirming the absence of toxic residues from the electrospun membrane processing. Moreover, the alignment of BMSCs in the aPCL and aPEC groups was observed to follow the orientation of the fibers, indicating that the fiber arrangement influences cell morphology (Figure 4A and B).

The impact of pECM on BMSC growth was assessed using the CCK-8 assay. Results revealed that PEC fibers coated with pECM significantly promoted BMSC proliferation compared to PCL fibers at all observed time points. Interestingly, no significant differences in promoting BMSC proliferation were observed between the random and aligned fiber morphologies (Figure 4C). These findings indicate that the electrospun membranes exhibit excellent cytocompatibility.

### Aligned pECM topology promotes osteogenesis *in vitro*

**Figure 5.**
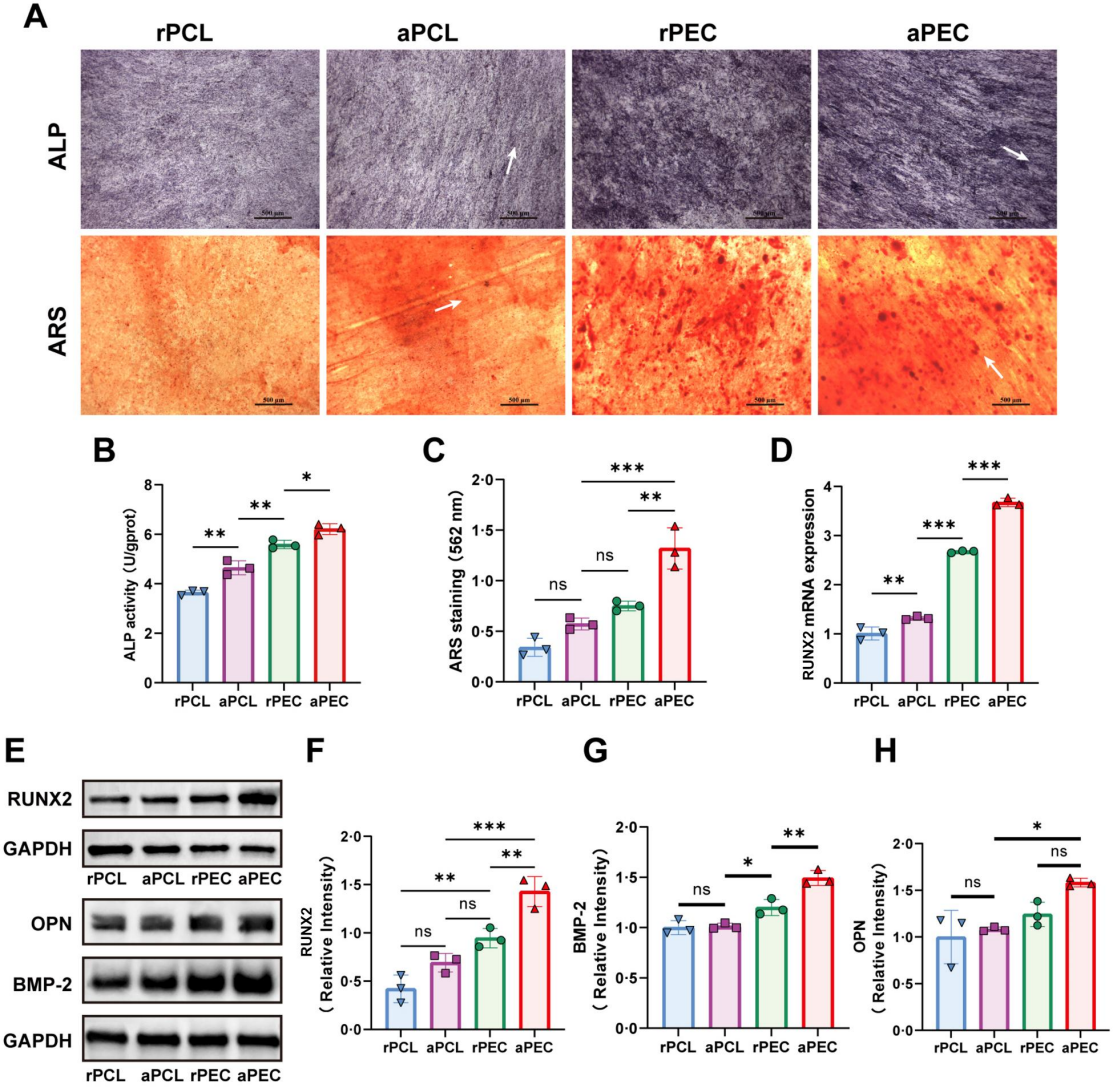
Aligned pECM topology promotes osteogenesis *in vitro*. (**A**) ALP staining on day 7 and ARS staining on day 14 during BMSC osteogenic induction. White arrows indicate the alignment direction of electrospun fibers. (**B**) ALP activity detection on day 7 during BMSC osteogenic induction. (**C**) Quantification of ARS staining. (**D**) qPCR detection of RUNX2 gene expression. (**E**) WB analysis of RUNX2, OPN, and BMP-2 protein expression in BMSCs after 7 days of culture. (**F–H**) Quantitative analysis of WB results. Data are presented as mean ± SD. ns, no significance, ***P < 0.001, **P < 0.01, *P < 0.05.

To assess the effect of topological structure on the osteogenic differentiation of BMSCs, ALP and ARS staining were performed. ALP staining revealed weak positive signals on PCL membranes, whereas markedly stronger signals were observed on PEC membranes. Since it was challenging to visually distinguish subtle differences in ALP staining between the aligned and random groups, ALP enzymatic activity was quantified. The results showed that the enzyme activity of the aPCL group (4.65 ± 0.29 U/gprot) was significantly higher than that of the rPCL group (3.64 ± 0.10 U/gprot). Similarly, the aPEC group (6.21 ± 0.22 U/gprot) exhibited significantly higher ALP activity compared to the rPEC group (5.59 ± 0.17 U/gprot) (Figure 5A and B).

ARS staining and quantification revealed significantly greater calcium salt deposition in the aPEC group compared to other groups. However, unlike ALP expression, ARS staining did not exhibit a significant difference between the aPCL and rPCL groups (Figure 5A and C). Interestingly, ALP- and ARS-positive regions in both the aPCL and aPEC groups were aligned with the orientation of the electrospun fibers, indicating that topology influences osteogenic matrix deposition (Figure 5A).

To further assess osteogenic differentiation, the gene expression of Runx2, a key osteogenic marker, was analyzed. The results revealed that Runx2 expression was significantly higher in the aPEC group than in the rPEC group (*P* < 0.001), with the aPCL group exhibiting a similar trend relative to the rPCL group (*P* < 0.01) (Figure 5D). WB analysis was performed to evaluate the protein expression of osteogenic markers in BMSCs cultured on different membranes. As shown in Figure 5E, the expression levels of RUNX2, BMP-2, and OPN were markedly elevated in cells cultured on aPEC membranes compared with aPCL and rPCL membranes. Quantitative analysis (Figures 5F–H) demonstrated that RUNX2 expression in the aPEC group was significantly higher than in the rPEC group (*P* < 0.01), indicating that the aligned pECM topology promotes osteogenic differentiation. Similarly, BMP-2 levels were significantly upregulated in the aPEC group compared with the rPEC group (*P* < 0.01) and other control groups, while OPN expression followed the same trend. Notably, no significant differences in the expression of any marker were observed between rPCL and aPCL groups.

These findings indicate that aligned pECM topology significantly enhances the osteogenic differentiation of BMSCs, whereas aligned PCL topology demonstrates limited efficacy in promoting osteogenesis.

### Aligned pECM topology promotes bone regeneration *in vivo*

**Figure 6.**
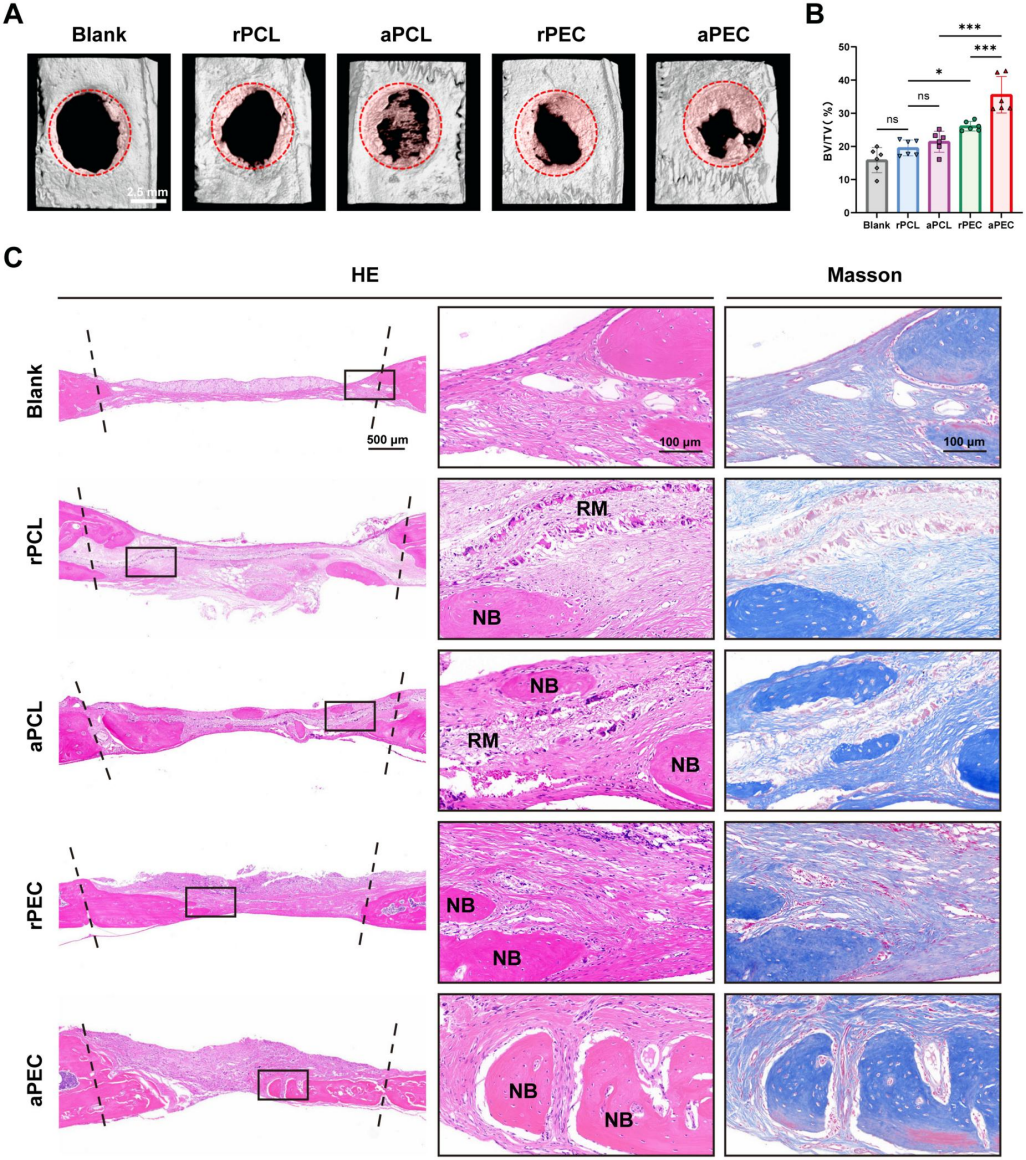
Aligned pECM topology promotes bone regeneration *in vivo*. (**A**) Micro-CT reconstruction images showing the material implanted into a 5 mm skull defect in rats for 4 weeks. Red circle indicates the defect region. (**B**) Quantitative analysis of new bone volume fraction based on 3D reconstructed micro-CT images. (**C**) HE and masson staining showing each group of membranes implanted for 4 weeks. Dotted line indicates the defect region. RM, residual material; NB, new bone. Data are presented as mean ± SD. ns, no significance, ***P < 0.001, **P < 0.01, *P < 0.05.

A rat critical-sized skull defect model was employed to evaluate the osteogenic potential of aligned pECM topology *in vivo*. Micro-CT reconstruction images at 4 weeks post-implantation demonstrated minimal bone tissue formation at the defect margins in the blank and rPCL groups. In the aPCL group, discontinuous, thin, cord-like regenerated bone spanned the defect area, whereas the defect region in the rPEC and aPEC groups was predominantly covered with new bone tissue. Notably, newly formed bone in the aPEC group extended centrally towards the defect site (Figure 6A). Quantitative analysis of bone volume fraction revealed a significantly higher proportion of new bone in the aPEC group (35.56 ± 5.48%) compared to the rPEC group (26.11 ± 1.42%). However, no significant difference was detected between the aPCL group (21.41 ± 3.15%) and the rPCL group (19.54 ± 2.37%) (Figure 6B).

Histological analysis using HE staining showed abundant connective tissue filling the defect region in the blank group. In the rPCL and aPCL groups, dense residual material persisted, accompanied by small, discontinuous new bone surrounded by connective tissue. Conversely, the rPEC and aPEC groups exhibited substantial material degradation, with large, continuous new bone replacing the defect region. Masson staining further confirmed that newly formed bone in the aPEC group exhibited greater maturity compared to other groups (Figure 6C).

These findings suggest that aligned pECM topology substantially enhances bone regeneration *in vivo*. In contrast, PCL topology demonstrates limited influence on *in vivo* osteogenesis.

### Aligned pECM topology enhances ITGB1 expression and promotes BMSC adhesion

**Figure 7.**
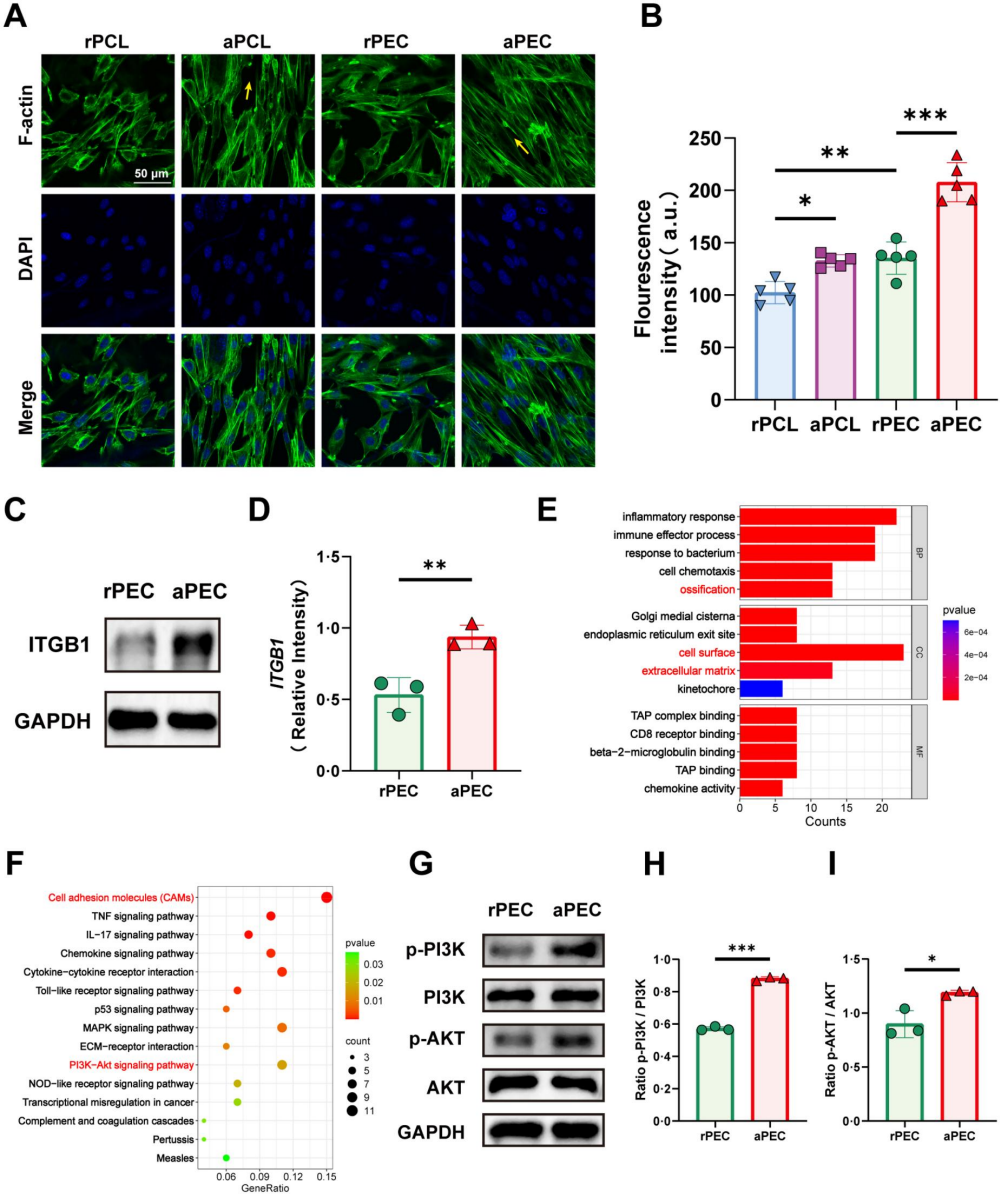
Aligned pECM topology activates the expression of ITGB1/PI3K/AKT signaling pathway. (**A**) Confocal microscopy images of BMSC adhesion. Green: F-actin, blue: DAPI. Yellow arrows indicate the direction of electrospun fiber arrangement. (**B**) Quantification of F-actin fluorescence intensity. (**C**) WB analysis to detect the expression of the key adhesion molecule ITGB1 and (**D**) quantitative data analysis. (**E**) GO enrichment analysis following sequencing of BMSCs cultured on rPEC or aPEC. BP: biological process; CC: cellular component; MF: molecular function. (**F**) KEGG pathway enrichment analysis. (**G**) WB analysis of protein expression levels of p-PI3K, PI3K, p-AKT, and AKT in BMSCs. Quantification of phosphorylation levels of (**H**) PI3K and (**I**) AKT. Data are presented as mean ± SD. ns, no significance, ***P < 0.001, **P < 0.01, *P < 0.05.

To evaluate the effect of aligned topology on BMSC adhesion, immunofluorescence staining of F-actin, a key cytoskeletal component, was performed on cells cultured on the four membrane types. As shown in Figure 7A, BMSCs on rPCL and rPEC exhibited polygonal morphologies with filopodia and lamellipodia extending in random directions, and actin filaments appeared disorganized. In contrast, cells on aPCL and aPEC displayed elongated spindle-shaped morphologies, extended pseudopodia, and abundant intercellular connections, with actin filaments and cytoskeletal structures oriented along the fiber alignment (yellow arrows). Notably, the effect of fiber alignment on BMSC orientation and cytoskeletal organization was more pronounced in the PEC groups than in the PCL groups, likely due to the bioactive cues provided by pECM. Quantitative analysis of F-actin fluorescence intensity confirmed significantly higher cytoskeletal organization in the aPEC group compared to all other groups (*P* < 0.001, Figure 7B). Based on these findings, subsequent experiments focused on rPEC and aPEC membranes to further investigate the role of pECM alignment in regulating cellular behavior.

WB analysis of the key cell adhesion molecule ITGB1 revealed that its expression was significantly higher in the aPEC group than in the rPEC group (*P *< 0.001). These results suggest that the aligned pECM topology enhances BMSC adhesion by increasing the expression of ITGB1 (Figure 7C and D).

### The expression of ITGB1 enhances the activation of the PI3K/AKT pathway

To further investigate the mechanism by which topology influences BMSC adhesion and osteogenic differentiation, transcriptome sequencing was performed on cells from the rPEC and aPEC groups. Gene Ontology (GO) enrichment analysis showed that the differential genes between the two groups were primarily related to functions such as ‘ossification’, ‘cell surface’, and ‘extracellular matrix’ (Figure 7E). Kyoto Encyclopedia of Genes and Genomes (KEGG) enrichment analysis indicated the activation of the Cell Adhesion Molecules (CAMs) and PI3K−Akt signaling pathways in the aPEC group (Figure 7F). Notably, ITGB1 serves as both a key component of CAMs and an initiator of the PI3K−Akt signaling pathway [23]. This suggests that the aligned pECM topology activates the downstream PI3K−Akt signaling pathway by increasing ITGB1 expression.

WB analysis was performed to assess the expression of key proteins in the PI3K−Akt pathway. The results demonstrated that the expression ratios of p-PI3K/PI3K and p-AKT/AKT were significantly higher in the aPEC group, confirming that the aligned pECM topology activates the ITGB1/PI3K/AKT signaling pathway, thereby promoting BMSC osteogenesis (Figure 7G–I).

### Aligned pECM topology regulates osteogenesis via the ITGB1/PI3K/AKT pathway

**Figure 8.**
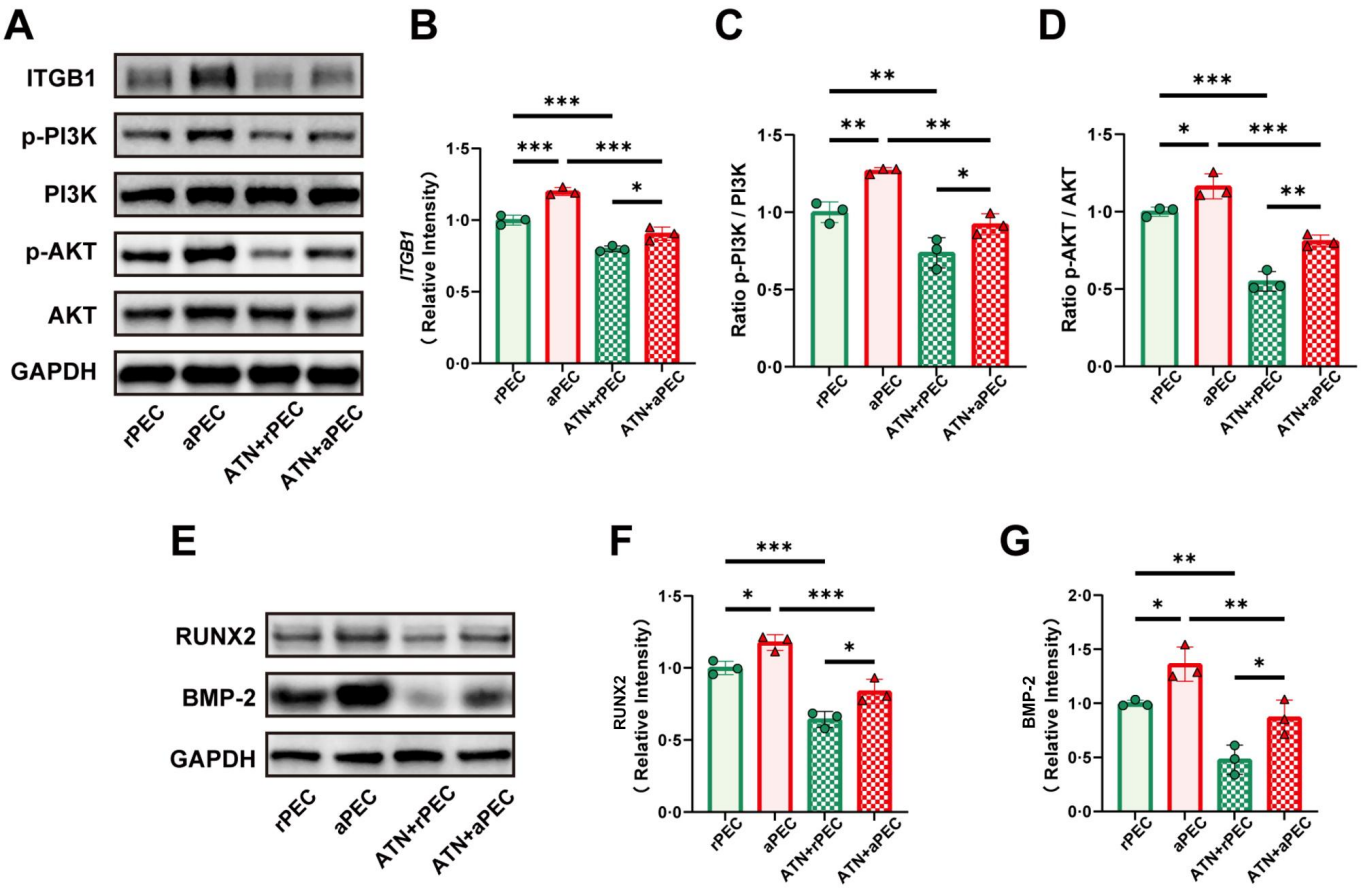
Inhibition of ITGB1 signaling attenuates the osteogenic effects of aligned pECM in BMSCs. (**A**) WB analysis of ITGB1, PI3K, p-PI3K, AKT, and p-AKT expression in BMSCs cultured on rPEC and aPEC membranes with or without the ITGB1 inhibitor ATN-161. (**B**) Quantification of ITGB1 expression. phosphorylation levels of (**C**) PI3K and (**D**) AKT. (**E**) WB analysis of osteogenic markers RUNX2 and BMP-2. Quantification of (**F**) RUNX2 and (**G**) BMP-2 expression levels. Data are presented as mean ± SD. ns, no significance, ***P < 0.001, **P < 0.01, *P < 0.05.

To investigate whether the aligned pECM topology mediates osteogenesis through the ITGB1/PI3K/AKT pathway, BMSCs cultured on rPEC and aPEC membranes were treated with the ITGB1 inhibitor ATN-161. As shown in Figure 8A, aPEC membranes significantly increased ITGB1 expression and enhanced PI3K and AKT phosphorylation compared to rPEC. Upon treatment with ATN-161, ITGB1 expression and the phosphorylation levels of PI3K and AKT were markedly reduced in both the ATN + rPEC and ATN + aPEC groups. Notably, ITGB1 expression and PI3K/AKT phosphorylation in the ATN + aPEC group remained higher than in the ATN + rPEC group, indicating that the aligned pECM topology promotes ITGB1 expression and subsequently upregulates the PI3K/AKT signaling pathway (Figure 8B–D).

Similarly, WB analysis revealed that aPEC significantly upregulated the expression of the osteogenic markers RUNX2 and BMP-2 compared to rPEC. Treatment with ATN-161 reduced the expression of these proteins in both the ATN + rPEC and ATN + aPEC groups. However, RUNX2 and BMP-2 levels in the ATN + aPEC group were still higher than those in the ATN + rPEC group (Figure 8E–G). These findings indicate that the aligned pECM topology enhances osteogenic differentiation of BMSCs by activating ITGB1 signaling and stimulating the PI3K/AKT pathway.

## Discussion

As the concept of bionics gains widespread recognition, the development of biomaterials with natural tissue topologies for skin, muscle, nerve, and bone regeneration holds significant promise [24–26]. Periosteal tissue possesses an aligned topological structure. Given the pivotal role of the periosteum in bone reconstruction, there has been growing interest in developing artificial periosteum with oriented bionic structures for bone regeneration [21, 27, 28]. However, the absence of research models based on periosteum-derived components has limited the exploration of specific mechanisms by which periosteum topology affects bone regeneration. In this study, a bionic periosteum with an aligned structure, derived from periosteum components, was fabricated using coaxial electrospinning technology, with pECM as the shell and PCL as the core, to investigate the impact of periosteum topology on bone regeneration. The results demonstrated that the aligned pECM promoted BMSC adhesion, enhanced osteogenic differentiation, and accelerated bone healing via the ITGB1/PI3K/AKT signaling pathway. This study elucidated the primary role of periosteum topology and clarified the underlying mechanisms influencing BMSC behavior, providing a foundation for designing bionic periosteum in future applications.

Artificial periosteum functions to isolate soft tissue in bone tissue engineering, provide adhesion sites for osteoblast-related cells, and facilitate cell growth under conditions mimicking natural environments [2, 29]. Additionally, it establishes a local microenvironment favorable for ossification while regulating cellular differentiation to repair damaged bone tissue. Existing reports offer diverse insights regarding the microtopological structure of artificial periosteum. Research by Albino Martins et al. reported comparable osteogenic gene expression in BMSCs cultured on random or aligned PCL electrospun membranes [12]. Similarly, Delaine-Smith et al. [13] found that aligned structures on gelatin-coated PCL electrospun membranes did not significantly enhance osteogenesis during its early stages, with similar findings reported by Kishore et al. [14] regarding aligned collagen lines. In contrast, several studies on hydrogels, hydroxyapatite, and collagen-coated PLA suggest that aligned artificial periosteal topologies promote osteogenesis effectively [8–10]. Cell adhesion to the surface of biomaterials is a prerequisite for subsequent cellular activities. In this study, cytoskeletal staining revealed that BMSCs cultured on membranes with an oriented topological structure exhibited elongated morphologies and well-aligned cytoskeletal organization along the fiber orientation. In contrast, cells on randomly structured membranes displayed polygonal shapes with disorganized actin filaments. Notably, the guiding effect of fiber alignment was more pronounced on oriented pECM than on PCL, which may be attributed to the bioactive components of pECM and the inherent hydrophobicity of PCL [30, 31]. Oriented pECM facilitated early BMSC adhesion and alignment along the fiber direction. Furthermore, ALP and ARS staining showed that the regions of positive staining corresponded to the orientation of pECM fibers. These findings suggest that the oriented topology of pECM may regulate the mineralization of the osteogenic matrix by modulating BMSC adhesion and differentiation.

KEGG pathway enrichment analysis identified several signaling pathways potentially associated with the observed effects, including integrin-mediated signaling. We focused on the ITGB1/PI3K/AKT pathway because ITGB1, a cell surface receptor, links the pECM to the actin cytoskeleton and transmits both chemical and mechanical signals to cells via adhesion complexes. This interaction with the ECM is critical for BMSC attachment, migration, and Runx2 phosphorylation. Activation of ITGB1 induces the expression of osteogenic markers in MSCs and promotes osteogenesis *in vivo* and *in vitro* by engaging the FAK/ERK and PI3K signaling pathways [32–36]. The experimental findings of this study indicate that aligned pECM activates the PI3K/AKT pathway downstream of ITGB1, which plays a pivotal role in regulating cytoskeletal functions, including cell adhesion, migration, and osteogenic differentiation [37, 38]. The PI3K/AKT pathway, in turn, stimulates the expression and activity of RUNX2 and orchestrates osteogenic differentiation through both direct and indirect mechanisms [39–41]. To further investigate the role of ITGB1 signaling, we conducted *in vitro* inhibition experiments using the ITGB1-specific inhibitor ATN-161. The results showed that blocking ITGB1 significantly reduced PI3K and AKT phosphorylation in BMSCs cultured on aligned pECM and downregulated the expression of osteogenic markers RUNX2 and BMP-2. Collectively, these findings suggest that the aligned topology of pECM enhances BMSC adhesion and osteogenic differentiation by activating the ITGB1/PI3K/AKT signaling pathway, thereby accelerating bone repair.

This study has several limitations. The effect of the periosteum’s aligned structure on BMSC migration could not be thoroughly investigated because the scratch assay, a primary method for evaluating cell migration, disrupts the aligned topology of the electrospun membrane, thereby compromising the reliability of the findings [42]. Additionally, due to the excellent hydrophilicity of pECM, it was not feasible to use specialized devices to confine cells to specific areas, as has been achieved in other studies [43]. Another limitation of this study is its focus solely on the effect of pECM on bone marrow-derived BMSCs, thereby limiting the investigation to a specific subset of cells responding to the periosteum’s aligned structure. Future studies should explore the influence of pECM on periosteum-derived cells to better elucidate the functional role of the periosteum in bone regeneration.

## Conclusion

This study developed a biomimetic periosteum characterized by an aligned structure derived from periosteum components and demonstrated that the aligned topological morphology accelerated bone healing compared to random pECM, enhancing BMSC adhesion and osteogenic differentiation through the ITGB1/PI3K/AKT signaling pathway. Additionally, it elucidated the primary functions and potential mechanisms of the periosteum’s topological structure, offering critical insights for advancing the design of biomimetic periosteum.
